# Non-small-cell lung cancer classification via RNA-Seq and histology imaging probability fusion

**DOI:** 10.1186/s12859-021-04376-1

**Published:** 2021-09-22

**Authors:** Francisco Carrillo-Perez, Juan Carlos Morales, Daniel Castillo-Secilla, Yésica Molina-Castro, Alberto Guillén, Ignacio Rojas, Luis Javier Herrera

**Affiliations:** Department of Computer Architecture and Technology, University of Granada. C.I.T.I.C., Periodista Rafael Gómez Montero, 2, 18014 Granada, Spain

**Keywords:** Deep learning, Gene expression, Late fusion, Whole slide imaging, NSCLC

## Abstract

**Background:**

Adenocarcinoma and squamous cell carcinoma are the two most prevalent lung cancer types, and their distinction requires different screenings, such as the visual inspection of histology slides by an expert pathologist, the analysis of gene expression or computer tomography scans, among others. In recent years, there has been an increasing gathering of biological data for decision support systems in the diagnosis (e.g. histology imaging, next-generation sequencing technologies data, clinical information, etc.). Using all these sources to design integrative classification approaches may improve the final diagnosis of a patient, in the same way that doctors can use multiple types of screenings to reach a final decision on the diagnosis. In this work, we present a late fusion classification model using histology and RNA-Seq data for adenocarcinoma, squamous-cell carcinoma and healthy lung tissue.

**Results:**

The classification model improves results over using each source of information separately, being able to reduce the diagnosis error rate up to a 64% over the isolate histology classifier and a 24% over the isolate gene expression classifier, reaching a mean F1-Score of 95.19% and a mean AUC of 0.991.

**Conclusions:**

These findings suggest that a classification model using a late fusion methodology can considerably help clinicians in the diagnosis between the aforementioned lung cancer cancer subtypes over using each source of information separately. This approach can also be applied to any cancer type or disease with heterogeneous sources of information.

**Supplementary Information:**

The online version contains supplementary material available at 10.1186/s12859-021-04376-1.

## Background

Nowadays, cancer is one of the most deadly diseases around the world just behind cardiovascular diseases. Providing early diagnosis tools has grown in interest over the last few decades. Lung cancer is one of the most frequent cancer types, with a total of 2.2 million new cancer cases and 1.8 million deaths worldwide in 2020 [1], representing the 18.0% of total cancer related deceases. Lung cancer is characterized by the uncontrolled cell growth in tissues of the lung organ. Most of the cancers that start in the lungs are carcinomas [2], and two main types can be differentiated within them: small-cell lung carcinoma (SCLC) representing around 15–20% of lung cancer cases, and non-small-cell lung carcinoma (NSCLC) representing around 80–85% of lung cancer cases [3]. Within NSCLC, two different subtypes can be differentiated, which are adenocarcinoma (LUAD) and squamous-cell carcinoma (LUSC). LUAD usually comes from peripheral lung tissue and it is usually associated with lifelong nonsmokers [4]. On the other hand, LUSC is closely correlated with a history of tobacco smoking, and tended to be more often centrally located [5, 6].

An appropriate identification of the NSCLC lung cancer subtype is critical in the diagnostic process, since therapies differ for LUAD and LUSC [7]. Therefore, finding accurate and robust biomarkers in different types of patient’s biomedical information is crucial to accelerate this process. Experts use several methods for lung cancer type classification, such as Computer Tomography screening, whole-slide imaging (WSIs), the identification of biomarkers in next-generation sequencing (NGS) data (e.g. gene expression analysis using RNA-Seq) or the use of clinical information from the patient. The manual analysis of these sources of information can be a time consuming and exhausting task. Thus, in recent years the automatic analysis of each of the aforementioned data types has been explored [8–12].

Using information fusion methodologies that integrate the predictions of systems using biological information may enhance the diagnosis of a patient. Information fusion has been a topic of interest in machine learning (ML) research in late years based on the growth of multimodal data. Three different approaches can be discerned depending on when the fusion takes place: early fusion, late fusion or, more recently presented, intermediate fusion [13]. In the early fusion methods a feature representation for all data modalities is obtained and then a concatenation of all is performed to serve as input to the final classifier [14]. On the other hand, late fusion methods train a separate model for each data modality using all the available training data for each of them on the given task. Once those models have been trained, the fusion model is created by fusing the outputs from each model to draw a decision, with the aim of improving the classification that each independent model is making [15]. At last, intermediate fusion is more linked to deep learning models. Here, the representations that have been obtained for the different modalities are fused in the middle layers of a deep neural network. The fusion of biological data has been mainly explored in literature for prognosis prediction, obtaining good results [16–23]. Since not all sources of information are always available, having an integration model for the classification would also allow to predict the lung cancer type even when only one source of information is available. A model of this characteristics would fall into the design of decision-making support systems that are applied to precision medicine [24], as the immediate future of bioinformatics and medicine. To the best of our knowledge, an integration of gene expression data and biomedical imaging to provide a classification model for LUAD, LUSC and healthy patients has not been proposed in literature.

The aim of this work is to present a classification model using a late fusion methodology for the task of LUAD, LUSC and healthy patients classification by fusing the probabilities obtained by two classifiers. One classifier uses as input RNA-Seq data and the other one WSI. In this section an introduction to the problem has been outlined. In the “Related work” section, an overview of the related works in the area of ML applied to lung cancer will be reported. In the “Methods” section, the methodology and data used for this work will be presented. In the “Results and discussion” section, results obtained for the proposed experiments will be shown and discussed. Finally, in the “Conclusions” section conclusions will be drawn and future work will be outlined.

## Related work

### Lung cancer gene expression classification

Over the last few years, the potential of ML models using NGS data for cancer classification has been shown. Specifically, several works can be found in literature for lung cancer type classification using gene expression data.

Since LUAD is the most frequent lung cancer type, many works have been published for LUAD and control classification. Smolander et al. presented a deep learning model using gene expression from coding RNA, and non-coding RNA [25]. They obtained a classification accuracy of 95.97% using coding RNA. Similarly, Fan et al. using Support Vector Machines (SVMs) and a signature of 12 genes reached an accuracy of 91% [26]. Zhao et al. combined the information from ncRNAs, miRNAs and mRNAs for the classification, using SVMs. Finally, they selected 44 genes and they reached a classification accuracy of 95.3% [27] .

For lung cancer subtypes classification, Gonzales et al. studied differentially expression genes (DEGs) between SCLC, LUAD, LUSC and Large Cell Lung Carcinoma. Then, different feature selectors and predictive models were used in order to compare their classification performance [28]. Authors reached an accuracy of 88.23% using k-NN and the Random Forest feature selector.

### Lung cancer histology imaging classification

In recent years, the use of deep learning models for histology imaging classification has been taken into consideration based on the outstanding results that these models have reached in computer vision tasks and in health informatics [29, 30]. Deep learning models require huge quantities of data in order to properly learn features from an image. Therefore, the most popular approach for histology image classification has been to perform a segmentation of regions of interest of each slide (or placing a label for the whole slide). Then tiles can be extracted and labeled by experts from the image for a posterior training. Thus, a huge increment in the available dataset is obtained.

Based on the aforementioned methodology, different works have been published for lung cancer classification. Coudray et al. used a deep learning model using transfer learning for LUAD, LUSC and control classification and mutation prediction, reaching a 0.978 score of area under the curve (AUC) [11]. Tiles were extracted and were used to perform the training and the classification. Similarly, Kanavati et al. presented a deep learning model using transfer learning for lung carcinoma classification, reaching a score of 0.988 AUC for a binary classification problem [31]. Authors used labeled images by experts for their work. Graham et al. presented a two steps methodology for LUAD, LUSC and normal classification using a deep learning model trained on image tiles and then extracting summary statistics from them for the final classification, obtaining an accuracy value of 81% [32]. Likewise, Yi et al. trained and compared the performance of several Convolutional Neural Networks (CNNs) with different structures in classifying image tiles as malignant vs. non-malignant, obtaining an AUC score of 0.9119 [33].

Yu et al. combined traditional thresholding and image processing techniques for slides images with machine-learning methods, achieving an AUC of  0.85 in distinguishing normal from tumor, and  0.75 in distinguishing LUAD from LUSC [34]. Khosravi et al. used deep learning for the classification of breast, bladder and lung tumors, achieving an AUC of 0.83 in classification of lung tumor types on tumor slides [35].

### Fusion of omics and histology data

When it comes to the integration of information from different omics data and histology imaging, different approaches have been proposed in the recent literature, mainly for prognosis prediction.

Lee et al. presented a multimodal longitudinal data integration framework based on deep learning to predict Alzheimer disease progression [19]. In this case, MRI scans, genomics information and cognitive assessments were used as inputs. In order to obtain the feature representation, a recurrent neural network with gated recurrent units [20] was used. Then, features were concatenated and a final prediction was performed.

Another methodology that has recently been presented in literature with remarkable results is obtaining a feature vector for each type of data and then performing a plain concatenation of those features vectors or applying attention mechanism before the concatenation for model training. Lai et al. developed a deep neural network (DNN) combining heterogeneous data sources of gene expression and clinical data to accurately predict the overall survival of NSCLC patients [21]. The combination of 15 biomarkers along with clinical data were used to develop an integrative DNN via bimodal learning to predict the 5-year survival status of NSCLC patients with high accuracy. The combination outperform the results obtained for each type of data separately. Silva et al. presented an end-to-end multimodal Deep Learning method, named MultiSurv, for automatic patient risk prediction for a large group of 33 cancer types [22]. They compared fusing different sources of information by using attention weights, and used a feed-forward neural network for predicting. Chen et al. presented an integrated framework for fusing histology and genomics features for survival outcome prediction [23]. The authors used WSIs, mutations information, Copy Number Variation (CNV) information and mRNAseq. Different features were obtained and fed to an attention mechanism, that was later used for survival prediction and grading. The results presented by the authors shown that the use of the fusion outperform the results obtained by using each type of data separately.

Finally, several works have been published where features used for classification have been obtained using autoencoders [36]. Cheerla et al. presented a deep learning model with multimodal representation for pancancer prognosis prediction [16]. The survival prediction of patients for 20 different cancer types was performed using as information clinical data, mRNA expression data, miRNA expression data and WSIs. Feature vectors were obtained and then combined for prognosis prediction. They obtain outstanding results, specially in those cancer types where not many samples were available. Simidjievski et al. investigated several autoencoder architectures to integrate a variety of patient data such as gene expression, copy number alterations and clinical data, showing the usefulness of this approach for different breast-cancer analysis tasks [17]. Following with the use of autoencoder architectures for information integration, Ma et al. proposed a Multi-view Factorization AutoEncoder (MAE) which not only encodes gene expression, miRNA expression, protein expression, DNA methylation and clinical information but also includes domain knowledge such as molecular integration networks for bladder urothelial carcinoma and brain lower grade glioma classification [18].

## Methods

### Data acquisition

In this work we have used two different types of biological data: RNA-Seq and WSIs. The data were gathered from the The Cancer Genome Atlas (TCGA) program [37], located in the GDC portal [38].

The TCGA contains information from 33 different cancer types, and they have achieved the goal of providing easy access to the data. In addition, GDC have performed an harmonization of all the available samples in the program. Moreover, various data types are available for each sample (e.g. gene expression, copy number variation, histology imaging, etc.). In GDC, each patient has a Patient ID that identifies them, and each screening performed on the same biological sample from a patient has a defined Case ID. Therefore, for each Case ID we can have different biological information (WSI, RNA-Seq or both). Those Case IDs used in this work are available in a Github repository that can be found in the “Availability of data and materials” section. Table 1 shows the Case IDs availability per class and considered data type.

**Table 1:** Number of samples per class for each data type

	WSI	RNA-Seq	Both
LUAD	495	457	442
Healthy	419	44	41
LUSC	506	479	467
Total	1420	980	950

WSIs data needs to be preprocessed prior to any analysis. The preprocessing of WSIs relied on the Python package openslide [39], that efficiently read and parse these type of images.

For the case of gene expression, RNA-Seq from Illumina HTSeq data is used in TCGA. In the specific case of GDC data, it harmonizes RNA-Seq data by aligning raw RNA reads to the GRCh38 reference genome building and calculating gene expression levels with standardized protocols [40]. The KnowSeq R-Bioc package was used in order to obtain the values of the DEGs [41].

Models were implemented in Python with the Pytorch [42] and Scikit-Learn [43] packages. Deep Learning model training was performed using a NVIDIA™ RTX 2080 Super Graphics-Processing-Unit (GPU).

In order to avoid a result bias due to a reduced test set and the data imbalance, the whole dataset was divided using a 10-Fold Cross-Validation (10-Fold CV), in order to obtain a more thorough assessment of the proposed methodology. In each iteration of the 10-Fold CV process, the training set was used to train the models, and also for hyperparameter tuning, while a final assessment of models performance was done in the test set. The hyperparameter tuning strategy used differs for each data type and will be later explained in each model section; concretely, a single traditional training-validation subdivision was used for the WSI model, and grid search CV was used for the RNA-Seq model. All the splits were performed both in a patient-wise way and in a stratified way. With a patient-wise splitting we are ensuring that, even if a patient has more than one case, they could only belong to one of the splits, being this training or validation. Imposing this restriction prevents any kind of information leakage during training. On the other hand, through stratified splitting the proportion of classes in each fold is maintained.

### WSI preprocessing

WSIs, also known as virtual microscopy, refers to scanning a complete microscope slide and creating a single high-resolution digital file. With it, different resolutions of the same image can be obtained and an extraction of tiles can be performed. The generated file has SVS format, and several preprocessing steps need to be taken in order to work with this type of files. Firstly, SVS images are read with an specific factor of magnification. In this work, a factor of 20 × was chosen in order to obtain an adequate resolution (leaving images with an approximate resolution of $10{,}000\times 10{,}000$). Once images were obtained, we converted them from SVS to PNG format in order to facilitate further manipulations.

We took non-overlapping tiles of 512x512 omitting those tiles where most of it was white background. To test this condition, we computed the mean value for each channel. If in all three channels the value was greater than 220, the tile was discarded, otherwise it was selected as proposed in literature by Coudray et al. [11]. This methodology allows to multiply the number of images available to train the model, since we are using all the tiles that can be extracted from each one of the images instead of only using the whole image. This enables the deep learning models to more easily learn relevant features for the classification task. The number of tiles per class is depicted in Table 2.

**Table 2:** Number of tiles per class

	# Tiles
LUAD	100,841
Healthy	62,715
LUSC	92,584
Total	256,140

### RNA-Seq data preprocessing

In order to analyze the HTSeq-Counts data provided, we used the KnowSeq R-Bioc package [41]. This package provides a pipeline to obtain DEGs based on the HTSeq-Counts files and then performs a machine learning assessment of the selected DEGs. KnowSeq relies on limma [44], which is the state-of-the-art for finding differential expressions. However, limma is usually employed to biclass problems, where two classes need to be compared. Thus, additional tasks need to be perform to achieve DEGs when there are more than two classes. In order to deal with this problem, Castillo et al. presented the coverage (*COV*) parameter, which uses limma to a perform binary comparisons of the *N* presented classes and finally select a set of genes that are differentially expressed in *COV* binary comparisons [45].

Therefore, we used the *DEGsExtraction* function from the KnowSeq package over the training set for obtaining the DEGs matrix. As parameters, a $Log_{2}$ *Fold Chain* (*LFC*) value of 2, a *p*-value of 0.05 and a *COV* value of 2 were set. Once we obtained the DEGs matrix, we used the minimun Redundancy Maximum Relevance (mRMR) algorithm to obtain a ranking of the genes [46]. The mRMR algorithm uses information theory for obtaining a ranking of features which are highly correlated with the classes but with a minimum redundancy between them. mRMR algorithm has been previously used in literature as feature selector for gene expression [45, 47–49].

### Foundations of the techniques used

In this work we have used two different ML algorithms: CNNs and SVMs.

Convolutional Neural Networks (CNNs) are a particular type of NNs that deal especially well with spatially correlated data (in the case of interest, 2D images) [50]. They use small filters that are applied to the data using a convolution operation. Many of these filters are applied and the results are stacked. Thanks to being localized in space, they can capture spatial patterns well, which make them especially useful for data such as images. In addition, by employing 1D convolutional layers useful temporal patterns can also be captured when working with different types of signals. The features that these filters produce can be later used to serve as input to other classifiers, or under an information fusion approach [16].

Kernel Methods (KMs), and more specifically SVMs [51, 52], are a very important family of learning algorithms. Their popularity arose in the mid 90s, and have been applied to a very large number of problems for decades. In nonlinear SVM classification, a maximum margin separating hyperplane is established in a dual space, so that the samples of different categories are divided by the widest possible gap. In case of non-separable data, a parameter called *Cost* (C) can be tuned, allowing a certain amount of errors to be made. By doing so, the generalization capabilities of the model can be optimized, to increase the performance on unseen data. Thus, new examples are then mapped and predicted to belong to a category based on which side of the gap they fall. Although on its basis, SVM is a binary classifier, it can also be applied as a multi-class classifier following an One-Against-One (OVO) set of classifiers methodology [53].

### Per-tile model and per-slide classification

For the per-tile classification we used a CNN, given that they have proven to be the state-of-the-art in computer vision problems, using a transfer learning approach. CNNs have been widely used in literature for WSIs classification with great results, as described in “Lung cancer histology imaging classification” section. In addition, given the size of the tile dataset, using other classical machine learning models might not be computationally feasible. Transfer learning allows to use the filters that have been learned in another problem domain with sufficient data, and adjust the weights of the network to another given problem. Different architectures were tested such as VGG-16 [54] or Efficientnet [55]. Finally, the Resnet-18 architecture [56] was used with pre-trained weights on Imagenet [57], and tiles were normalized with the mean and standard deviation from it. The classification layer of the architecture was adapted to the set of classes, but preserving the same structure. Only the last residual module of the architecture was fine-tuned, the rest of the weights of the network were frozen. As it is usual for deep learning models, in each split a 10% of the training data was used as validation set for the network optimization and hyperparameter tuning

For the CNN training 25 epochs were used, monitoring the accuracy on the validation subset with the early stopping methodology and saving the best weights for later use. As loss function, the cross entropy loss function was selected. As the optimizer, Adam was chosen with a learning rate (LR) value of $1e^{-5}$, betas equal to (0.9, 0.999) and epsilon equal to $1e^{-8}$. Since the Resnet-18 is being fine tuned, a small LR need to be used or the pre-trained weights will change more than desired. These hyperparameters were chosen by manually tuning them during the experimentation, and based on results in the validation set.

Once we have obtained a per-tile model, now we need to define how to classify a slide. In this work we used a majority voting approach, similar to the methodology presented by Coudray et al. [11]. Having all tiles from the image classified using the per-tile model, the most predicted class among all the tiles is used as the final prediction. Variations using using different thresholds (instead of simple majority voting) to choose the final prediction were inspected. Nevertheless, in our case the aforementioned methodology provided the best performance.

### RNA-Seq model

For the RNA-Seq feature extraction, we carried out the preprocessing steps explained in “RNA-Seq data preprocessing” section. Selecting how many genes to perform the classification with is of utter importance, since usually clinicians are looking for the smallest gene signature that led to a good classification performance. This decision is important due to the necessity of providing a small gene signature that can facilitate its use in a standard clinical laboratory, for instance in a PCR-based diagnosis assay [58–60]. We used three different sets of genes (3, 6 and 10) in order to compare the performance of the fusion model when using a comparatively small, medium and large size of gene signatures.

It is important to note, that for the simulations performed under the 10-Fold CV assessment, it implies using different training datasets for the gene signatures extraction. This could lead to small variations in the signatures obtained [61], since we are using a different group of samples as training set each time. The final gene signature proposed was the one formed by those genes that are best ranked by the mRMR algorithm in the rest of gene signatures.

We tested different classification techniques for the RNA-Seq classification task such as K-Nearest Neighbors, SVMs or Random Forest. Finally, SVMs were selected, since they outperformed the rest of the models in the validation set and they have proven to be really successful in mid-size problems. In addition, it has been used in the gene expression literature for cancer classification with good results [45, 47, 48]. A grid search CV was used over the training set in each split for the parameters optimization, using the Gaussian Radial Basis Function kernel as it has proven to offer a good asymptotic behavior [62]. The search range of values for both *C* and $\gamma$ was: $[2^{-7}, 2^{-5}, 2^{-2}, 2, 2^{4}, 2^{7}]$. Moreover, features were normalized between $-\,1$ and 1.

### Probability fusion

As it has been reported in the “Background” section, among the different approaches proposed in the literature for data fusion, this work uses a late fusion methodology. We also performed experiments using early fusion approaches, in which obtained features from both RNA-Seq and WSIs data types were concatenated and fed to a classifier performing the final prediction. Under this last scheme, the straightforward features extracted for each data type (gene expression on one side, and accumulation -average sum- of the features extracted from the CNN for the different tiles of an image) was observed to decrease the performance of the fusion classification model. This decrease in the performance in comparison to the late fusion model may be due to the difference between the dimensionality of the features obtained from each data type, since a feature vector of size 512 is obtained in the case of the WSI and a feature vector of size between 3 and 10 genes is obtained in the case of RNA-Seq. Even after applying a number of approaches to reduce the feature dimensionality, such as PCA or maxpooling operation on the CNN features, results were not surpassing the late fusion scheme next explained.

There exist two options when applying a late fusion approach: to combine the predictions or to combine the probabilities returned by each classifier. These two approaches rely on the classification models used. The first option, i.e. integrating the predictions, would require to weight the classifiers, since only two data types are used. The second, the fusion of the probabilities, allows including more information for the classification (i.e. the probabilities assigned by each classifier to each class). So this last option was selected in expectancy of a more powerful information fusion.

The probabilities for each classifier were obtained as follows. For the RNA-Seq model, the probabilities are returned by the SVM by using the methodology proposed by Wu et. al. [63], which uses a pairwise coupling method that is is included in the Scikit-Learn library [43]. With this methodology we are able to obtain three probabilities, one per class, which model the belonginess of a sample to each class, and where the sum of the probabilities is equal to one. On the other hand, for the WSI we need to compute them. In the per-slide classification we have the number of tiles predicted per class, therefore, we compute the probability per class for each slide as: the number of tiles predicted for a class divided by the total number of tiles in the slide (see Eq. 1).

1 $$\begin{aligned} P^{^{CNN}}{(x,c_i)} = \frac{\#TilesPredicted_{(x,c_i)}}{\#SlideTiles} \end{aligned}$$

where *x* is the sample to be predicted and $c_i$ is the given class: LUAD, Healthy or LUSC. Using this methodology we are able to obtain three probabilities (one for each class) representing how likely is for that slide to belong to each one of the classes, depending on the predictions provided by the CNN. In addition, and given that the number of predicted tiles per class are divided by the total number of tiles, the sum of the obtained probabilities is equal to one.

Once we have obtained the probabilities for each classifier and class, we need to fuse them to make a final prediction. In this work we propose a weighted sum of the two probabilities by using two weight parameters: $\alpha _{1}$ and $\alpha _{2}$ (see Eq. 4). They will control the trade-off between the probabilities returned by the two models: $\alpha _{1}$ for the WSI CNN classifier ($P^{^{CNN}}$) and $\alpha _{2}$ for the RNA-Seq SVM classifier ($P^{^{SVM}}$). This will allow both classifiers to support each others predictions: in case one of the classifiers is providing a borderline wrong decision, the other one could balance it to the right side.

Some approaches have been proposed in literature to weight the probabilities obtained from classifiers using different modalities. Dong et al. proposed to give a weight to each classifier based on the performance of each model [64]. Similarly, Meng et al. proposed to compute the weight based on the accuracy of each model applying a normalization between the maximum and the minimum accuracy [65]. Trong et al. proposed to normalize the accuracy only based on the maximum accuracy achieved in order to obtain a weight [66]. Other approaches have been taken, such that proposed by Depeursinge et al. where the the probabilities returned by two SVMs were multiplied and the maximum was chosen for the prediction [67].

In this work, the weight for each classifier is computed based on their mean performance in ten different stratified resampling sets obtained from the training set. Resampling is a methodology that consists on taking a random subset of samples from a given set, usually a percentage of it, and has shown to be useful for robust statistic estimation [68]. In this work, a 90% of the training set was randomly chosen for each resampling in a stratified way, i.e. maintaining the percentage of each class in each set. Due to the imbalance of the dataset, the F1-Score metric was chosen as performance measure. Thus, firstly the mean of F1-Score metric is obtained across the ten different resamplings of the training set as follows:

2 $$\begin{aligned} {\overline{F1}}_{M} = \frac{\sum _{i=1}^{10} F1_{M_{i}}}{10} \end{aligned}$$

where ${\overline{F1}}_{M}$ is the mean F1-Score of a model *M* (i.e., *CNN* or *SVM*) across all the resampling sets and $F1_{M_{i}}$ is the F1-Score metric obtained by the model *M* in the *ith* resampling set.

Then, after computing the mean of the F1-Score for each model across the 10 different resampling sets, the final weight for each classifier is computed as follows:

3 $$\begin{aligned} \alpha _{1}= \,& {} \frac{{\overline{F1}}_{CNN}}{{\overline{F1}}_{CNN} + {\overline{F1}}_{SVM}} \nonumber \\ \alpha _{2}= \,& {} \frac{{\overline{F1}}_{SVM}}{{\overline{F1}}_{CNN} + {\overline{F1}}_{SVM}} \end{aligned}$$

where ${\overline{F1}}_{CNN}$ and ${\overline{F1}}_{SVM}$ are the mean F1-Score obtained by each model across the resampling sets, and it is satisfied that $\alpha _{1} + \alpha {2} = 1$.

These $\alpha _{1}$ and $\alpha _{2}$ are then used for the fusion model in order to weight the probability returned by each classifier. Thus, the probability for a sample *x* belonging to a class $c_i$ will be calculated using the following equation:

4 $$\begin{aligned} P^{^{Fusion}}{(x,c_i)}= \alpha _{1} * P^{^{CNN}}{(x,c_i)} + \alpha _{2} * P^{^{SVM}}{(x,c_i)} \end{aligned}$$

**Fig. 1:**
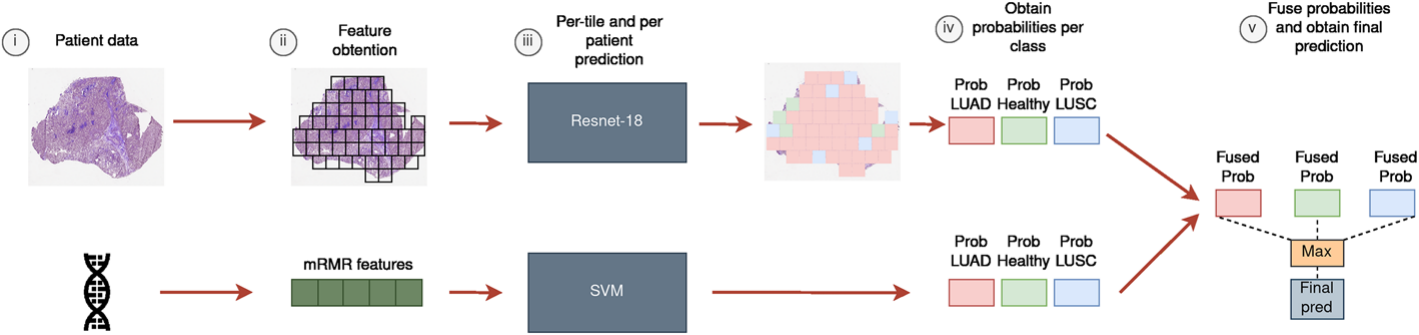
Pipeline for sample prediction. (i) Both the WSI and the RNA-Seq data for that case ID are obtained. (ii) Non-overlapping $512\times 512$ tiles are extracted for the WSI, filtering the background. For RNA-Seq, we took the set of DEGs selected by the mRMR ranking. (iii) For the WSI, the probabilities are obtained by averaging the number of tiles predicted per class and the total number of them. For RNA-Seq data, the probabilities are returned by the SVM. (iv) We fuse the probabilities by averaging the ones obtained by each classifier per class, and the final prediction is the class with the higher probability

With this automatic methodology we are getting a weight value for the classification that allows to fuse the classifiers’ probabilities based on how well they performed on the training set. Given that we are using a 10-Fold CV for evaluating the methodology, different $\alpha$ values might be obtained for each split, since different training sets are being used. It is important to note that this fusion methodology allows to effectively deal with missing information. If one of the data types is missing, then only the probabilities of the other classifier will be taken into account, without the need to average them. The pipeline for estimating the probability a given sample belonging to the class is depicted in Fig. 1

## Results and discussion

The presented results are for those cases where both sources of information are available (see Table 1), allowing a fair comparison of the improvement that can be obtained under the information fusion approach. Models were trained on all the available data in each training set, and global assessment is presented using a 10-Fold CV approach on the whole dataset. All results are presented for the CNN per-slide classification using WSI as input data, the SVM using RNA-Seq data, and the fusion model. We computed the accuracy, F1-Score, confusion matrices, ROC curve and Area Under the Curve (AUC) for each set and split.

Table 4 shows the accuracy, F1-Score and AUC for the WSI classifier, the RNA-Seq classifier and the fusion model. Results are averaged for the ten executions and the standard deviation obtained is also shown.

With respect to the RNA-Seq classifier, three different set configurations were tested: 3, 6 and 10 genes. The RNA-Seq model obtains good results across the splits for the three configurations, with relevant improvement observed when using 6 genes over 3. However a very similar performance is observed when using 10 genes (94.05% of F1-Score, and 94.12% of accuracy) in comparison with 6 (93.67% of F1-Score, and 93.70% of accuracy), even with a higher standard deviation when using 10 genes. This enables to choose a gene expression model with 6 genes without significantly affecting the performance in comparison to using a larger gene set, which facilitates its utilization in a standard clinical laboratory [58, 59]. For the AUC metric, the model also achieves impressive results with both sizes, reaching 0.987 and 0.990 respectively. These results are comparable to those obtained for a binary classification problem (LUAD vs Healthy) where authors reached an accuracy of 95.97% and 91% (see “Lung cancer gene expression classification” section).

In relation to the WSI data classification, Table 4 shows that this model presents a lower classification performance in comparison to the RNA-Seq and fusion model, achieving an F1-score of 83.39% and an accuracy of 86.03%. For the AUC metric, the results are similar or even improve in some cases those obtained in literature (see “Lung cancer histology imaging classification” section), achieving 0.947 in the validation set across the splits.

The fusion model was optimized using the methodology proposed in the “Probability fusion” section, choosing an optimized value of $\alpha _{1}$ and $\alpha _{2}$ for each split. It must be noted that the range of the $\alpha$ values obtained in each configuration was very similar across the splits, with $\alpha _{1}$ ranging from [0.49–0.52] and $\alpha _{2}$ ranging from [0.48–0.51]. The fusion model outperforms the RNA-Seq and WSI models for all metrics (see Table 4). The configuration of the fusion model using 3 genes is slightly outperformed by the RNA-Seq model using 6 genes. This is due to the lower performance of the RNA-Seq model configuration when using 3 genes. However, the fusion model still achieves a better performance in comparison to the WSI and the RNA-Seq configuration using 3 genes (see Table 4). Taking the configuration with 6 genes, the fusion model achieves a mean F1-Score of 95.19%, a mean AUC of 0.991 and a mean accuracy of 95.18%. For that model, Figs. 3 and 4 show the confusion matrices and the ROC curves for the whole dataset. For the fusion model, similar results are obtained when using 6 and 10 genes, which allows to use the model with a smaller gene signature. Given the low number of healthy samples where both data types are available (see Table 1), it is interesting to note that the mean F1-Score achieved is high, which means that these are being correctly classified on the whole dataset. The standard deviation of the metrics across the splits decreases with the fusion model, showing that it allows a more stable behaviour than the separate SVM and CNN models. The results obtained in the classification problem are also comparable to those obtained in literature, reaching those accuracies obtained in a binary classification problem when using RNA-Seq data (95.97 % [25], 91% [26], 95.3% [27]) and the AUC obtained when using WSIs as input for the multi-class classification (AUC 0.978 [11]) and for the binary classification (AUC 0.988 [31]).

In order to visualize the performance of each model per class, we plotted the ROC curves for the whole dataset (see Fig. 4), for the fusion model with 6 genes. Confusion matrix was also extracted for the whole dataset (see Fig. 3). As it can be observed, the fusion of probabilities obtains a better performance for the three classes over the CNN and the SVM models. In addition, the fusion model reduces the number of missclassified samples from 133 and 60 to 46, for the CNN and the SVM respectively, over the whole dataset when using 6 genes (see Table 5). This represents an improvement of the error rate of $\approx 65\%$ over the CNN, and $\approx 24\%$ over the RNA-Seq model.

**Fig. 2:**
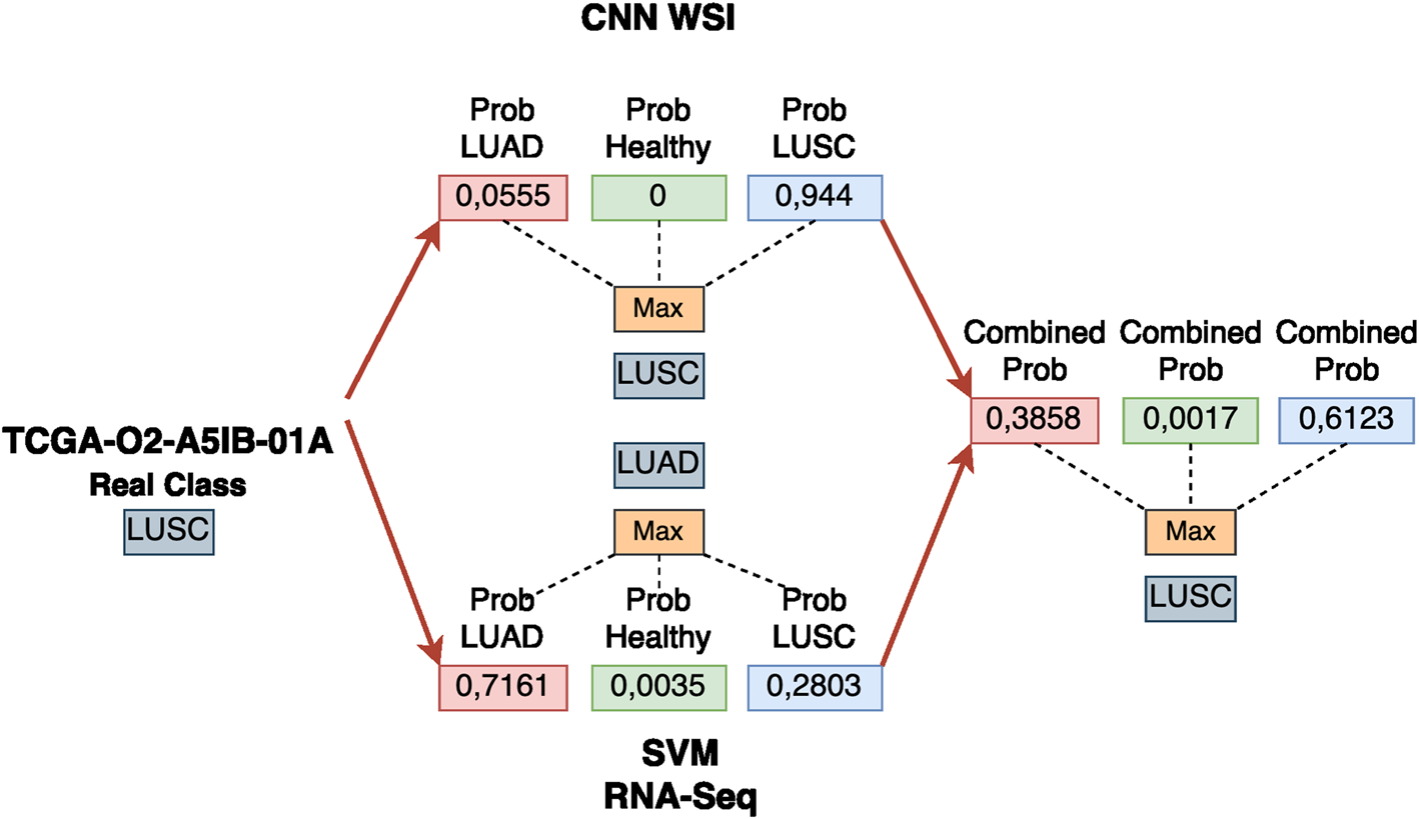
Example of the correct classification of a specific sample ID combining the probabilities. In the example shown, RNA-Seq classifier is providing a certain level of uncertainty between LUAD and LUSC classes, and due to the clear confidence of the CNN model for the LUSC class, the outcome of the fusion model provides the right diagnosis

**Fig. 3:**
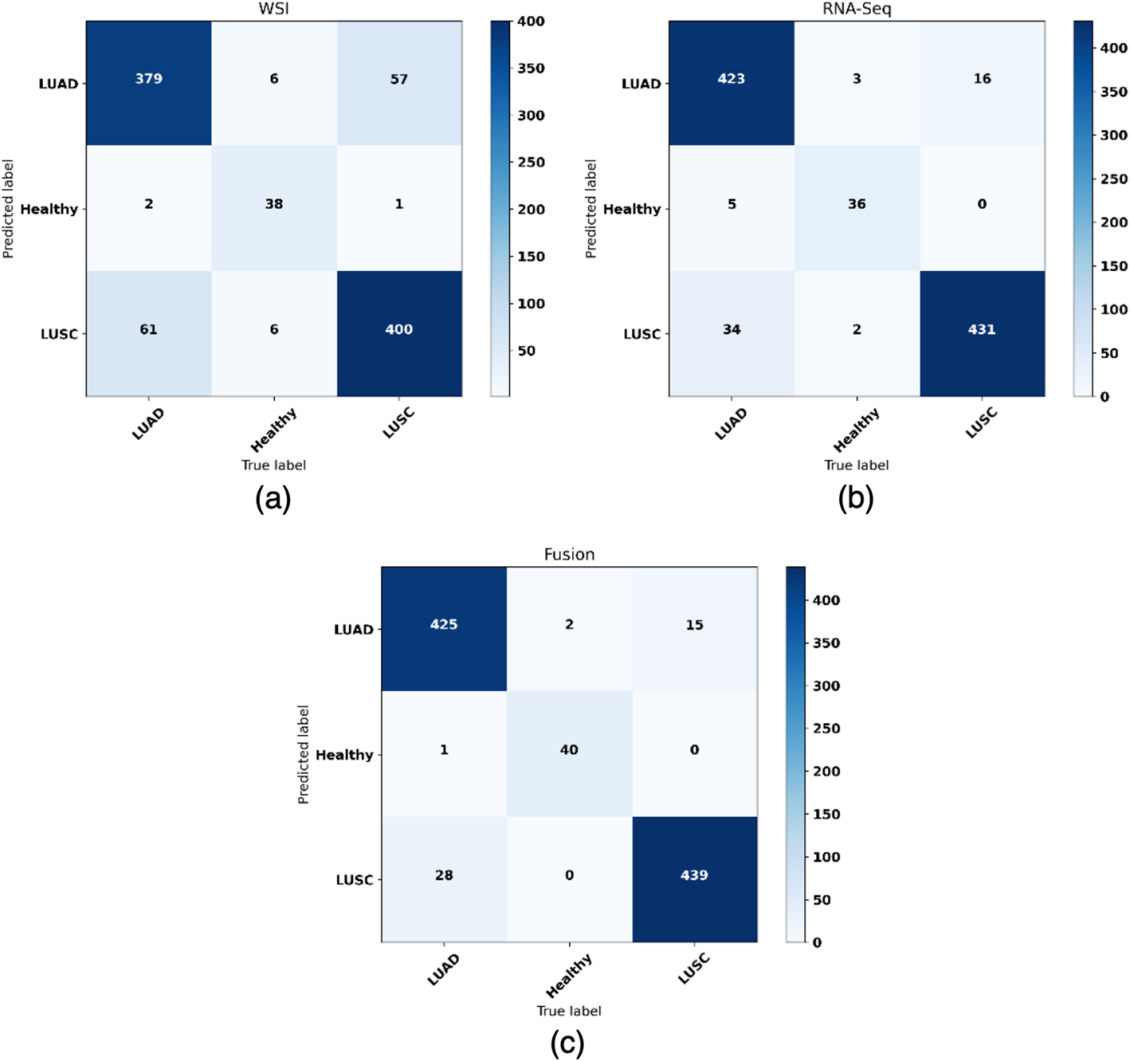
Confusion matrices obtained for the validation set in the 10-Fold CV by, **a** the CNN using WSI, **b** SVM using RNA-Seq data using 6 genes, **c** the fusion model using 6 genes. The accuracy and the f1-score is displayed under each confusion matrix

Based on the results we have obtained, the fusion model is correctly classifying samples that one of the models was wrongly predicting (see Fig. 3 and Table 5). We analyzed models’ predictions for the whole dataset to assess the cases in which both classifiers were providing different outcomes. An example can be observed in Fig. 2.

Finally, in order to provide a biologically relevant single gene signature for clinical use, the use of a single gene signature was inspected. As the final unique gene signature, we selected the one from the ten obtained in the 10-Fold CV process whose genes appeared in the first positions of the mRMR ranking for the rest of the splits. The 6-genes signature is formed by the following genes: *SLC2A1,NTRK2,TOX3,NXPH4,TFAP2A,KRT13*. The correlation of these genes with lung cancer was verified in the Open Targets platform [69], whose association scores with cancer, lung cancer, NSCLC, LUAD and LUSC, are shown in Table 3. Its performance was evaluated over the 10-CV, achieving a mean F1-Score, AUC and accuracy of 94.35%, 0.985 and 94.32% respectively for the isolate RNA-Seq SVM model, and 95.31%, 0.991 and 95.29% for the fusion model. In addition, a biological relevance analysis of these DEGs can be found as Additional file 1.

**Table 3:** Final Association Scores for the DEGs selected by mRMR

mRMR Genes	Cancer	Lung Cancer	NSCLC	LUAD	LUSC
SLC2A1	0.53	0.27	0.30	0.08	–
NTRK2	1.0	0.30	0.30	0.06	0.04
TOX3	1.0	0.11	0.09	0.07	–
NXPH4	–	–	0.14	–	–
TFAP2A	1.0	0.68	0.70	0.67	-
KRT13	0.80	0.03	0.10	–	0.01

These scores have been obtained using the Open Targets platform [69]

**Table 4:** Mean accuracy, F1-Score, AUC and standard deviation (in parenthesis) across the 10-Fold CV validation splits for each data type

	F1-Score (%)	AUC	Acc. (%)
WSI	83.39 (8.19)	0.947 (0.023)	86.03 (3.40)
RNA-Seq 3	90.57 (3.66)	0.978 (0.009)	90.67 (3.73)
RNA-Seq 6	93.67 (1.76)	0.987 (0.007)	93.70 (1.87)
RNA-Seq 10	94.05 (2.51)	0.990 (0.005)	94.12 (2.56)
Fusion 3	93.20 (3.18)	0.986 (0.005)	93.20 (3.17)
Fusion 6	95.19 (1.64)	0.991 (0.004)	95.18 (1.64)
Fusion 10	95.18 (1.61)	0.991 (0.005)	95.17 (1.62)

**Table 5:** Correct and erroneous predictions across the 950 samples when using 3, 6 and 10 genes

	WSI	RNA-Seq 3	Fusion 3
Correct	817	861	885
Misclassified	133	89	65

	WSI	RNA-Seq 6	Fusion 6
Correct	817	890	904
Misclassified	133	60	46

	WSI	RNA-Seq 10	Fusion 10
Correct	817	894	904
Misclassified	133	56	46

**Fig. 4:**
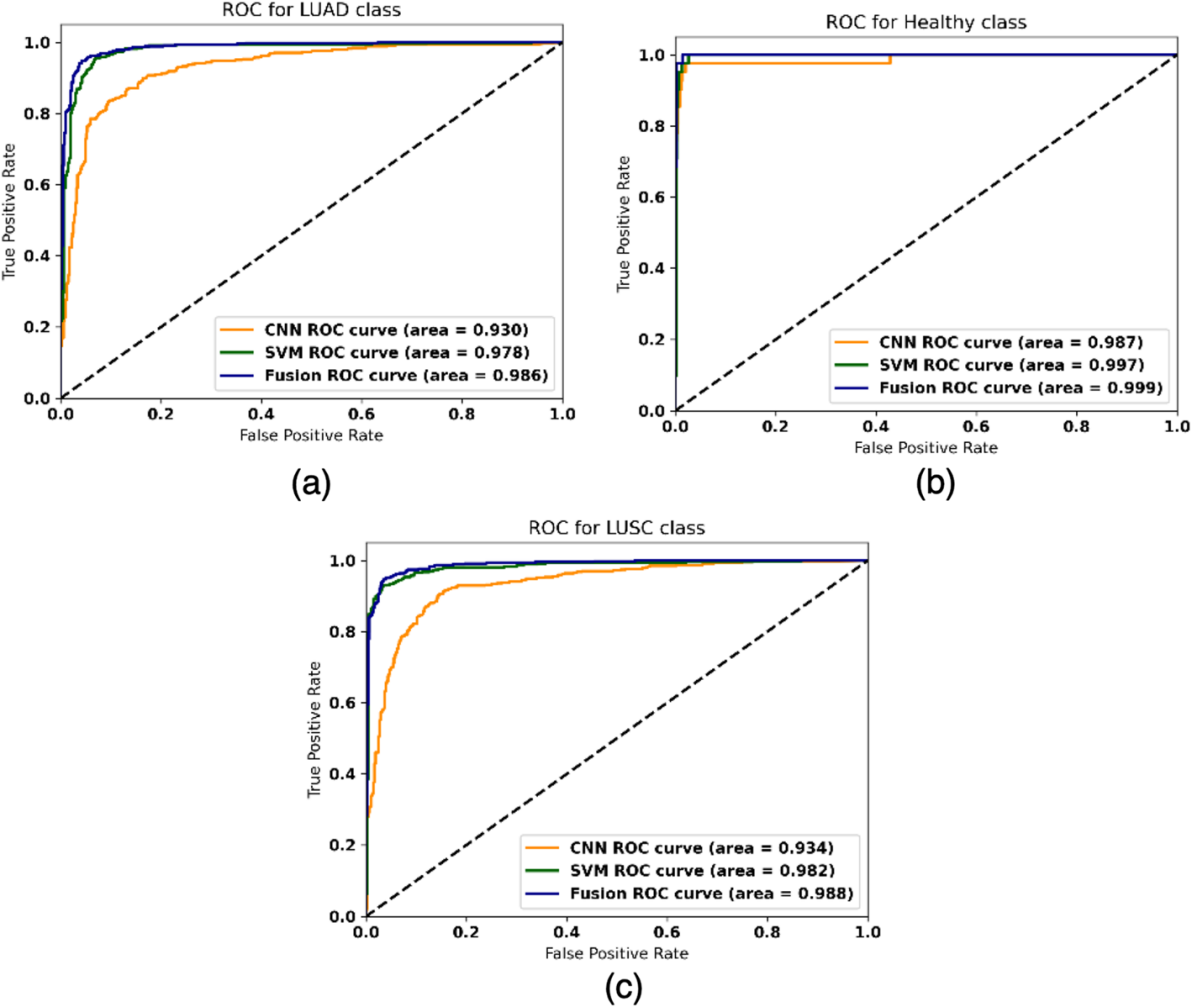
ROC curves obtained for the validation set in the 10-Fold CV for the CNN using WSI, SVM using RNA-Seq data using 6 genes, the fusion model using 6 genes for **a** LUAD, **b** Healthy and **c** LUSC classes. The Area Under the Curve for each classifier is displayed in the legend

## Conclusions

In this paper, we have proposed a late fusion classification model using RNA-Seq and WSIs data for LUAD, LUSC and healthy classification.

Promising results are obtained with each source of information, showing their potential to find cancer biomarkers. However, the proposed late fusion approach outperforms the results obtained by each classification model using RNA-Seq and WSIs in an isolate manner. It also reaches a more stable classification performance as observed in the experiments. This fusion model not only allows to integrate the predictions from each classifier but also enables a prediction when some of the information is missing. The methodology used can also be universally applied to any kind of problem with heterogeneous data that presents missing information and the modularity of the system makes it easily scalable, so new classifiers for different types of data can be integrated with little effort.

The presented methodology represents an advancement in the creation of decision-making support systems that are applied to precision medicine, which can be used in a real-life scenario. With the integration of different sources of information, a more robust and complete prediction can be performed, similarly to those situations in an hospital where different screenings are performed in order to diagnose a patient. A quick detection of any type of cancer in its early stage is crucial to improve the survival of the patient. Hence, accurate and fast methodologies, such as the one presented, can enhance the treatment of the patient.

As future work, we would like to test the proposed methodologies on other cancer types or diseases, in order to evaluate its general applicability. In addition, we would like to include more heterogeneous biological sources and domain knowledge, extending the flexibility of the model in face of real scenarios with different screenings performed, in expectancy of an increase in the liability of the global diagnosis support system.

## Supplementary Information

**Additional file 1.** Biological relevance analysis of the selected gene signature.

## Data Availability

Those Case IDs used in this work and the implementation will be available in the following Github repository upon acceptance of this work: https://github.com/pacocp/NSCLC-multimodal-classification.

## Abbreviations

NGS: Next generation sequencing
TCGA: The Cancer Genome Atlas
GDC: Genomic data commons
DEGs: Differential expressed genes
COV: Coverage
mRMR: Minimum redundancy maximum relevance
SVM: Support vector machines
CNN: Convolutional neural network
WSI: Whole-slide imaging
LFC: Log fold change
NSCLC: Non-small-cell lung cancer
LUAD: Lung adenocarcinoma
LUSC: Lung squamous cell carcinoma
AUC: Area under the curve
