# Supplementary material for: Non-small-cell lung cancer classification via RNA-Seq and histology imaging probability fusion
Source: BMC Bioinformatics. 2021 Sep 22;22:454. doi: 10.1186/s12859-021-04376-1 (PMC8456075; doi:10.1186/s12859-021-04376-1)
Supplement: Supplementary file 1 — Additional file 1. Biological relevance analysis of the selected gene signature. [file 12859_2021_4376_MOESM1_ESM.pdf]

Supplementary Material

Biological relevance of DEGs

The six DEGs found in our study were involved in different biological processes, usually associated with tumor development and cancer progression. Glucose metabolism can be found among these altered functions. Since cancer cells are highly proliferative, hypoxia often occurs when tumor cells outstrip their vasculature. As a consequence, cancer cells show enhanced glycolysis as a means for energy production; this phenomenon is known as 'Warburg effect' [1, 2]. *SLC2A1*, also called *GLUT1*, codes for a glucose transporter that facilitates glucose transport across the plasma membrane [3]. *SLC2A1* overexpression was found in a series of solid tumors, including lung cancer [4]. More specifically, *SLC2A1* overexpression has been reported in bronchial brushing samples of NSCLC patients, and *SLC2A1* expression in NSCLC tissues was higher than than in adjacent tissues [3]. In addition, *SLC2A1* overexpression has been linked to a poor prognosis in different cancer types, including NSCLC [5].

Some of the regulated genes encode for structural proteins, suggesting that cell architecture is also altered in NSCLC, which is related to loss of cell adhesion, invasiveness, migration and metastasis. *KRT13* gene encoded for a keratin (keratin-13), an intermediate filament protein expressed by epithelial cells in a cell-specific and differentiation-dependent manner [6]. It seems that enhanced *KRT13* expression in squamous-cell carcinomas could disturb the functions of cytoskeleton-cell junction desmosome and hemidesmosome protein complexes, consequently affecting cell adhesion and cell architecture, and indirectly affecting tumor behavior, neuroendocrine phenotypes, epithelial–mesenchymal transition (EMT) and stemness [7].

Modulation of genes that participate in signaling pathways such as *NTRK2* and *NXPH4* was also detected in our study. *NTRK2* is a member of the neurotrophic tyrosine kinase genes that encode for one of the Trk family proteins, TkrB. The NTRK family plays a role differentiation and maturation of the central and peripheral nervous system through activation of the PI3K-AKT and MAPK signaling pathways. However, *NTRK* gene fusions are found in solid tumors as oncogenic fusions responsible for growth and proliferation of cancer cells [8, 9]. Several studies have indicated that TkrB overexpression is oncogenic in several malignant tumors, including lung cancer. It might also be correlated with lymph node metastasis, vascular invasion and poor survival. Interestingly, Ozono et al. suggested that the binding of TkrB to one of its ligands, BDNF, promotes proliferating migratory and invasive phenotypes and cellular plasticity in LUSC [10], as previously reported for LUAD [11]. In contrast, although *NXPH4* up-regulation has been suggested in LUSC, its contribution to the disease remains unknown [12].

Furthermore, several of the DEGs were transcription factors, such as *TFAP2A and TOX3*. *TFAP2A* codes for the AP-2 transcription factor and many studies have been described it is markedly up-regulated, both in LUSC and LUAD tissues, compared with normal lung tissues. It seems correlated with poor prognosis, particularly among smokers [13], and it has been implicated in cancer proliferation, invasion, angiogenesis and EMT. Previous studies reported that *TFAP2A* promotes EMT by regulating TGF- signaling in cancer cells and regulates tumor growth via hypoxia inducible factor-1a (HIF-1a) signaling in nasopharyngeal carcinoma and

NSCLC [14]. For the *TOX3* gene, the protein produced contains an HGM-box, indicating that it may be involved in bending and unwinding of DNA and alteration of chromatin structure. Although its function remains unclear, it may be involved in various DNA-dependent processes [15]. TOX is a novel gene family that serves a pivotal function in human immunity. Recently, deregulated expression of TOX family members has been reported in a wide range of human cancer types. Notably, *TOX3* expression has been reported to be significantly increased in LUAD, compared with other pathological subtypes of lung cancer. Survival analysis demonstrated that elevated *TOX3* expression is significantly associated with improved progression-free and overall survival in patients with LUAD [16].

Author details

References

1. Tan Z, Yang C, Zhang X, Zheng P, Shen W. Expression of glucose transporter 1 and prognosis in non-small cell lung cancer: a pooled analysis of 1665 patients. Oncotarget. 2017;8(37):60954.
2. Smolle E, Leko P, Stacher-Priehse E, Brcic L, El-Heliebi A, Hofmann L, et al. Distribution and prognostic significance of gluconeogenesis and glycolysis in lung cancer. Molecular oncology. 2020;14(11):2853–2867.
3. Zhao H, Sun J, Shao J, Zou Z, Qiu X, Wang E, et al. Glucose transporter 1 promotes the malignant phenotype of non-small cell lung cancer through integrin β1/Src/FAK signaling. Journal of Cancer. 2019;10(20):4989.
4. Koh YW, Lee SJ, Park SY. Differential expression and prognostic significance of GLUT1 according to histologic type of non-small-cell lung cancer and its association with volume-dependent parameters. Lung Cancer. 2017;104:31–37.
5. Yu M, Yongzhi H, Chen S, Luo X, Lin Y, Zhou Y, et al. The prognostic value of GLUT1 in cancers: a systematic review and meta-analysis. Oncotarget. 2017;8(26):43356.
6. Li Q, Yin L, Jones LW, Chu GC, Wu JB, Huang JM, et al. Keratin 13 expression reprograms bone and brain metastases of human prostate cancer cells. Oncotarget. 2016;7(51):84645.
7. Nguyen TQ, Hamada A, Yamada K, Higaki M, Shintani T, Yoshioka Y, et al. Enhanced KRT13 gene expression bestows radiation resistance in squamous cell carcinoma cells. In Vitro Cellular & Developmental Biology-Animal. 2021:1–15.
8. Rolfo C, Raez L. New targets bring hope in squamous cell lung cancer: neurotrophic tyrosine kinase gene fusions. Laboratory investigation. 2017;97(11):1268–1270.
9. Wong D, Yip S, Sorensen PH. Methods for identifying patients with tropomyosin receptor kinase (TRK) fusion cancer. Pathology & Oncology Research. 2020;26(3):1385–1399.
10. Ozono K, Ohishi Y, Onishi H, Nakamura K, Motoshita J, Kato M, et al. Brain-derived neurotrophic factor/tropomyosin-related kinase B signaling pathway contributes to the aggressive behavior of lung squamous cell carcinoma. Laboratory Investigation. 2017;97(11):1332–1342.
11. Zhang S, Guo D, Luo W, Zhang Q, Zhang Y, Li C, et al. TrkB is highly expressed in NSCLC and mediates BDNF-induced the activation of Pyk2 signaling and the invasion of A549 cells. BMC cancer. 2010;10(1):1–8.
12. Zhang F, Chen X, Wei K, Liu D, Xu X, Zhang X, et al. Identification of key transcription factors associated with lung squamous cell carcinoma. Medical science monitor: international medical journal of experimental and clinical research. 2017;23:172.
13. Cheng C, Ai Z, Zhao L. Comprehensive analysis of the expression and prognosis for TFAP2 in human lung carcinoma. Genes & Genomics. 2020;42:779–789.
14. Lu J, Liu Rq, Hu Mj, Zhao Ym, Tan S, Wang Sy, et al. Chromatin accessibility analysis reveals that TFAP2A promotes angiogenesis in acquired resistance to anlotinib in lung cancer cells. Acta pharmacologica Sinica. 2020;41(10):1357–1365.
15. Hsu YL, Hung JY, Lee YL, Chen FW, Chang KF, Chang WA, et al. Identification of novel gene expression signature in lung adenocarcinoma by using next-generation sequencing data and bioinformatics analysis. Oncotarget. 2017;8(62):104831.
16. Zeng D, Lin H, Cui J, Liang W. TOX3 is a favorable prognostic indicator and potential immunomodulatory factor in lung adenocarcinoma. Oncology letters. 2019;18(4):4144–4152.
